# A fast and efficient size separation method for haploid embryonic stem cells

**DOI:** 10.1063/1.5006326

**Published:** 2017-10-31

**Authors:** Remo Freimann, Anton Wutz

**Affiliations:** Institute of Molecular Health Sciences, Swiss Federal Institute of Technology, ETH Hönggerberg, Otto-Stern-Weg 7, 8093 Zurich, Switzerland

## Abstract

Hemizygous mutations introduced in haploid genomes can directly expose a phenotype, thus facilitating gene function analysis and forward genetic screening. Recently, mammalian haploid cells could be derived from mouse, rat, monkey, and human embryos and have been applied to screens of cellular mechanisms including cell signaling, pathogen host factors, and developmental pathways. Notably, haploid cell cultures have an intrinsic tendency for diploidization and, thus, require periodic cell sorting. Here, we report a method for rapid purification of haploid mouse embryonic stem cells from mixed cell populations with high viability and yield. Our method uses membranes with micrometer pores for force-free separation and facilitates enrichment of haploid cells without flow cytometry. The separation method simplifies maintaining haploid cell cultures and has further applications in establishing haploid cell lines from embryos and isolating cell cycle phases of mammalian cells.

## INTRODUCTION

Forward genetic screening contributes to understanding the genome function and evolution. Thereby, phenotypes of haploid (ha) cells can be analyzed readily due to the lack of compensation for hemizygous gene mutations. Haploid genetics has been extensively performed in microbes such as yeast for gaining insights into molecular pathways.^1^ Haploid early developmental stages of normally diploid (di) animals can also be generated by micromanipulation or activation of eggs.^2,3^ Haploid embryonic stem cell (haESC) cultures have been derived from haploid mouse,^4^ rat,^5^ and monkey^6^ embryos and most recently human partenogenotes.^7^ The potential for genetic modification has facilitated the application of haESC lines for genetic screening by the means of transposon and viral^8^ gene trap vectors or chemical mutagenesis.^9^ In addition, gene modifications have been introduced in a hemizygous state using CRISPR/Cas based methods for isolation of homozygous cell lines.^8,10^ These approaches have been used to identify pathogen mechanisms, cellular pathways, gene essentiality, and targets of drug mechanisms.^9–12^ Although haESCs can be cultured similar to diploid embryonic stem cells (diESCs), an intrinsic self-diploidization tendency necessitates periodic purification of haploid cell cultures after a certain time or number of passages. Generally, enrichment of haploid cells is achieved by fluorescence activated cell sorting (FACS). Thereby, staining with DNA intercalating fluorochromes (typically Hoechst 33342) facilitates the sorting of cells with a single genome (1n) DNA content. The necessary instrumentation, setup, and staining procedures require time and effort, presently limiting the work on haploid mammalian cells. The use of DNA intercalating agents can furthermore increase spontaneous mutation rates and influence chromatin organization,^13,14^ which is undesirable for genetic investigations. For this reason, methods for preventing diploidization have been investigated. There is evidence that a prolonged metaphase of haESCs is associated with self-diploidization,^15^ and it could be shown that accelerating mitosis can stabilize haESCs to some degree.^15–17^ Recently, chemical inhibition of ROCK and CDK1 kinase activity has been reported to suppress diploidization and facilitate the establishment of differentiated cells with a haploid genome.^18^ Despite this progress, diploidization cannot be entirely prohibited and cell sorting remains essential for deriving and culturing haESCs.

Here, we present a new method for maintaining haESC cultures without the need for DNA staining or FACS. Based on the finding that haESCs are phenotypically smaller than diESCs,^7,18^ purification of haESCs from a cell mixture can be achieved by applying a force-free separation using membranes with defined micrometer sized pores, which have been calibrated to allow haploid but not diploid cells to pass through.^19,20^

## MATERIALS AND METHODS

### Construction and usage of the separation device

**FIG. 1.**
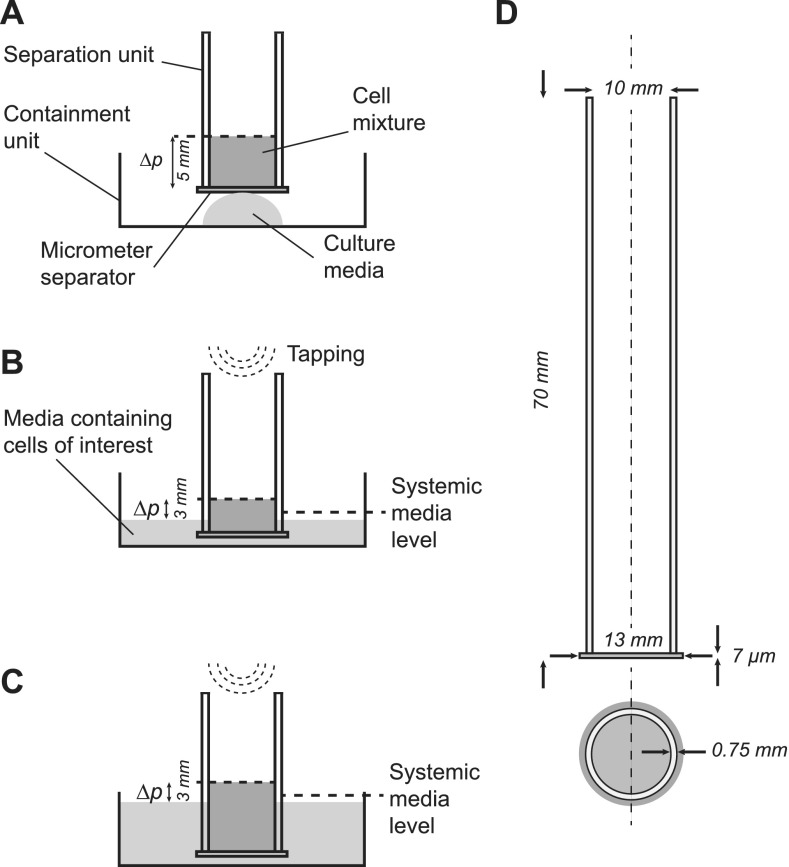
Schematic overview of cell separation. (a) A small amount of the starting cell mixture is loaded into the separation unit, and flow is initiated by contact with the media in the containment unit. (b) Agitation of the suspension by tapping increases the yield. (c) The hydrostatic pressure differential Δ*p* is kept small by submerging the separation unit in the medium as more cells are loaded. (d) Detailed reference implementation of the separation unit.

We constructed separation units from a polycarbonate track etch membrane (PCTE, Sterlitech) attached to the bottom of an open vessel (5 ml polystyrene tubes, Falcon) using a two-component epoxy adhesive (2-K-Epoxidkleber, UHU). Membranes with aperture sizes of 5, 8, 10, and 12 *μ*m were used for different experiments. A containment unit [12-well plate, Nunc, Thermo Fisher Scientific, Fig. 1(a)] was used to collect the purified cells. For obtaining viable haploid cells after passing through the micrometer aperture, external forces including forces induced by pressure, vacuum, or centrifugation must be avoided. Separation is performed by layering 0.4 mL medium containing cells onto the separation unit, whereby the hydrostatic pressure is kept low [Fig. 1(a), see Video 1 in the supplementary material]. A small amount of media is placed into the containment vessel. Through the contact of the membrane with the medium, passage of cells is initiated [Fig. 1(a)]. Importantly, the relative hydrostatic pressure is compensated by immersing the separation unit into the medium of the containment [Figs. 1(b) and 1(c)] to avoid mechanical damage to the cells. Cells are loaded incrementally, whereby the fluid level in the separation unit should not exceed 3 mm above the containment media level [Figs. 1(b) and 1(c)]. Gentle tapping of the top of the separation unit induces vertical movement of 1 to 2 mm, thereby agitating the cell suspension and reducing the chance of clogging of the pores [Figs. 1(b) and 1(c); Video 1 in the supplementary material]. Tapping at a frequency of 1 to 3 per second is continued until the cell suspension passed through and subsequent aliquots are loaded. The purified haploid cells are collected from the containment unit. To increase the yield, one or two additional elutions with cell culture media can be applied (Fig. 2 in the supplementary material).

**FIG. 2.**
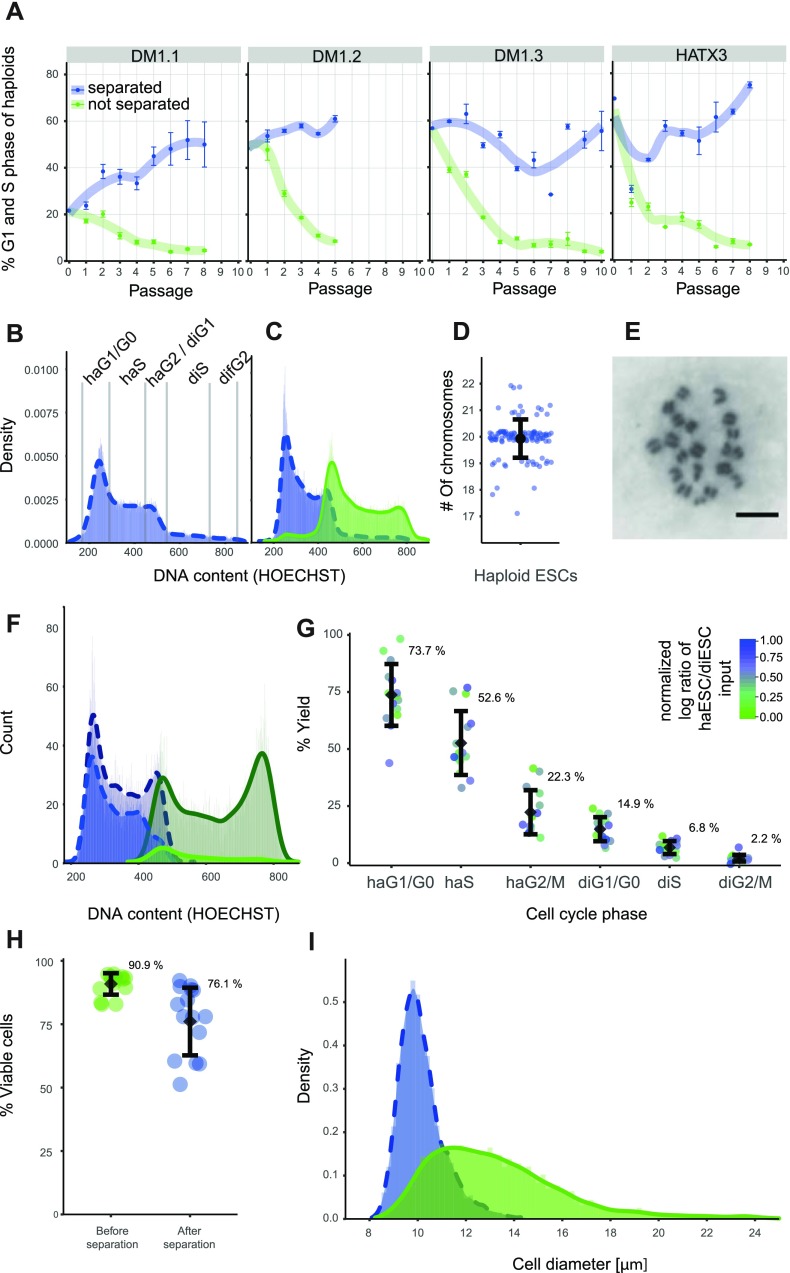
Cell separation performance of 8 *μ*m pores. (a) Haploid G1 and S phase fractions from DNA profiles of 4 cell lines (DM1, DM1.2, DM1.3, and HATX3) over several passages using the separation unit (blue) or not (green). Error bars represent the standard deviation (n = 3). (b) and (c) Histograms and fitted density curves of the DNA content. Cell cycle phases (G1, S, and G2/M) of haploid and diploid cells are indicated. (c) Analysis of HATX3 cells before (b) and after 8 passages (c) using the separation unit (blue bars, dotted line) or not (green, solid line) is shown. (d) Chromosome numbers by metaphase spreads of HATX3 cells after 6 passages using the separation unit (n = 115; modal chromosome number: 20). (e) Representative image of a metaphase spread. The scale bar represents 20 *μ*m. (f) DNA profiles of a 1:1 mixture of haESCs (dark blue bars, dotted line) and EGFP marked diESC (dark green, solid line) before and after separation (haESC: blue bars, dotted line and diESC: green, solid line). (g) Yield for cell cycle phases as indicated (n = 15). Colors indicate the feature scaled log transformed haESC/diESC ratios of the input cell suspension. (h) Cell viability of cell populations as measured by PI exclusion before and after separation. The mean (black dot) and standard deviation (error bars) are indicated. (i) Cell size distribution as measured by microscopy image analysis of a mixture of haESCs and diESCs (4:6) before (green) and after (blue) separation.

### Cell culture

Derivation and expansion of haESC cell lines from Xist^TX/TX^ R26^nlsrtTA/nlsrtTA^ (HATX3)^4,8^ and 129S6/SvEvTac mice (DM1.1-3) were performed, as previously described.^4^ After expansion on irradiated mouse embryonic fibroblast feeders, cells were sorted with a MoFlo Astrios EQ cell sorter (Beckman Coulter). The haG0/G1 peak was selected after staining with 15 *μ*g ml^−1^ HOECHST 33342 (Thermo Fisher Scientific) for 20 min at 37 °C. Sorted cells were subsequently expanded in a chemically defined 2i medium plus leukemia inhibitory factor (LIF) as described before.^21^ Cryostocks were prepared after one to four passages after sorting and transferred to liquid nitrogen storage. All experiments were subsequently performed in serum and LIF containing media: DMEM-high glucose (Thermo Fisher Scientific), 15 vol. % fetal bovine serum (PAN Biotech), glutamine, Pen/Strep, NEAA, sodium pyruvate (Thermo Fisher Scientific, 1 vol. % each), 8 *μ*l l^−1^ β-mercaptoethanol (Merck), 2000 U ml^−1^ LIF (homemade), 1.5 *μ*M CHIR99021 (Gene-operation), and 0.5 *μ*M PD0325901 (Gene-operation). Cells were cultured in 12-well plates (Thermo Fisher Scientific) that were coated with a 0.2% solution of gelatin (Sigma) and feeders.

### Application of the separation device: Time course experiment

Four independent cell lines consisting of different ratios of haploid and diploid cells [from 20% to 70% haESC, Fig. 2(a)] were thawed from cryostorage and cultured for up to 10 passages. Cell cultures were split and passaged every 2 days either using the separation device or without. Briefly, cells were washed twice with phosphate-buffered saline (PBS, pH 7.4; Life Technologies) for 2 min and incubated with a 0.25% trypsin/1 mM EDTA solution (Thermo Fisher) for 4 min at 37 °C. A single cell suspension was prepared by mechanical rocking of the plate, inactivation of trypsin by adding the serum containing medium, and pipetting several times before centrifugation at 1000 m s^−2^. The supernatant was discarded, and the cells were resuspended in 2 ml serum and LIF media. 0.5 ml aliquots were analyzed to assess the haploid cell content by flow cytometry. An aliquot of the cell suspension was then plated into a new dish either without purification or using the separation unit (n = 3 for each treatment).

### Separation performance experiment

Enhanced green fluorescent protein (EGFP) expressing HATX3 cells were generated using the pPB-CAG-EGFP transposon, and a CAG-PBase transposase vectors as previously described.^22^ Briefly, cells were transfected with Lipofectamine 2000 (Thermo Fisher) following the manufacturer's instructions and flow sorted 24 h thereafter for the diS/diG2/M fraction. An additional round of sorting was used to obtain isogenic purely diploid EGFP labeled cells. Haploid HATX3 cells were sorted for the haploid G1 and S phases. Mixtures of freshly FACS purified haploid haHATX3 and EGFP expressing diploid diHATX3 cells (haESC:diESC ratios of 0.06, 0.11, 0.18, 0.29, 0.36, 0.6, 0.99, 1.24, 1.66, 1.74, 3.32, 4.01, 7.04, 10.67, and 43.61) were prepared and subsequently passed through the separation unit to assess the separation characteristics. Quantification of the input (haESC/diESC ratio before separation) and the output (haESC/diESC ratio after separation) was performed by flow cytometry collecting absolute count data standardized to the volumes of the input and the output. Hoechst 33342 and EGFP signals were used to obtain separate DNA profiles for haHATX3 and diHATX3 cells. The yield [Fig. 2(g)] was calculated as $\frac{{\#\text{cells}}_{\text{output}}}{{\#\text{cells}}_{\text{input}}}$ × 100% for each cell cycle phase independently.

### Cell viability measurement

The viability of cells was assessed for different cell mixtures (n = 15) before and after the passage through the separation device. Cells were stained with 15 *μ*g ml^−1^ HOECHST 33342 for 20 min at 37 °C in a cell culture medium, and 4 *μ*g ml^−1^ propidium iodide was added for 5 min (PI, Thermo Fisher Scientific) prior to flow cytometric analysis. For comparison, cell viability was also assessed for flow sorted cells.

### Flow cytometry

Data were acquired using a MoFlo Astrios EQ cell sorter (Beckmann). DNA profiles were measured using a 100 mW 355 nm laser (Xcyte, attenuation level D) and a 448/59 nm band pass filter. GFP signals were detected using a 165 mW 488 nm laser (Coherent) and a 526/52 nm band pass filter. PI was measured using a 200 mW 561 nm laser (Coherent) with a 620/29 nm band pass filter. Sorting gates for embryonic stem cells (ESCs) were set from forward scatter (FSC) and side scatter (SSC) signals acquired with the 488 nm laser line using the Kaluza 1.2 Software (Beckman Coulter), and singlets were gated by using the SSC signal width. The cell cycle phases have been assessed from DNA profiles [Fig. 2(b)] and were separated using the EGFP signal of diESC to determine separation performance [Fig. 2(f), green histograms correspond to EGFP positive cells]. The gating strategy is shown in Fig. 1 in the supplementary material.

### Cell size distribution

The cell size distribution was assessed before and after passaging through the separation device. Cells were collected in a 6-well plate (Thermo Fisher Scientific). Bright field images of 8533 separated and 6101 not separated cells were obtained using a Zeiss Axio Observer Z1 microscope (Carl Zeiss AG) at 20-fold magnification. A MATLAB (MathWorks) script was used to measure cell sizes.

### Karyotyping

HATX3 cells were passaged 6 times using the separation device. Cells were then trypsinized as described before and passed through a 30 *μ*m cell strainer (Sysmex Partec) to obtain a single cell suspension. After centrifugation, cells were resuspended in a mixture of medium and ultrapure water (1:1 volume ratio) for 15 min at room temperature and subsequently fixed in ice-cold methanol:acetic acid (3:1 volume ratio) for 30 min at −20 °C. After replacing the fixative, the cells were incubated over night at 4 °C. Spreads were prepared by dropping the cells onto a moisturized slide. Slides were air dried and stained with Giemsa for 20 min before microscopy analysis.

## RESULTS

We previously determined that the cell size of haploid mouse ESCs is distinctly smaller and clearly separated from that of diploid cells.^18^ This prompted us to attempt purification using suitably sized porous materials. Polycarbonate track etch membranes are commercially available with pore sizes between 5 and 20 *μ*m which appeared suitable to separate haploid from diploid mouse ESCs. To experimentally investigate this possibility, we assembled a simple separation device [Fig. 1(a)] and used it in a typical filtration setup. Briefly, 1 ml cell suspension was pipetted into the tube and the flow through collected. However, using this procedure essentially, no viable cells could be obtained. Neither haploid nor diploid cells grew out after plating, indicating that passage through micrometer pores is not compatible with cell survival.

To reduce mechanical forces, we next applied small aliquots of cell suspension and collected cells that passed through the membrane in a reservoir filled with the medium. Preliminary testing indicated that viable cells can be obtained when pressure and forces can be kept at a minimum. Cell type specific membrane aperture sizes were determined by experimentally testing a range of aperture sizes around an estimate threshold size obtained by our earlier cell diameter measurements.^18^ Cell size measurements by microscopy imaging were previously performed for FACS sorted haG1, haG2, diG1, and diG2 phase cells with cell diameters measuring between 10.9 *μ*m and 16.7 *μ*m for haG1 and diG2 cells, respectively.^18^ Practically, the aperture size for obtaining a high purity of haploid cells is considerably smaller than the estimated cell size due to inherent cell deformability. Evaluation of separation units using polycarbonate filters with aperture sizes of 5, 8, 10, and 12 *μ*m showed that 8 *μ*m pores resulted in a high yield and purity of haESCs, whereas smaller apertures damaged the cells and reduced the yield of haploid cells. Larger pores led to a sharp increase in diploid contaminating cells. Using 8 *μ*m purification, a high proportion of haESCs could be maintained over time, whereas conventional passaging without enrichment led to a sharp increase in diploid cells, consistent with previous reports [Figs. 2(a), 2(b), and 2(c)]. A virtually complete separation of the haploid cells form mixed cultures can be obtained by tapping the separation unit while sequentially loading small amounts of cell suspension followed by cell culture media (Video 1 and Fig. 2 in the supplementary material). An intact haploid chromosome set was observed in the large majority of metaphase spreads from cells that have been repeatedly purified using the separation device [Figs. 2(d) and 2(e)]. These data demonstrated that purification through micrometer pores is compatible with haploid ESC cultures and can be used to effectively enrich for haploid cells in mixed cultures.

We then characterized the separation performance of 8 *μ*m pores in detail. For distinguishing haploid and diploid cells in cell mixtures, we generated isogenic EGFP labeled diploid ESCs from HATX3 cells by auto-diploidization and cell sorting. Defined mixtures of haploid and EGFP labeled diploid ESCs were then prepared and passed through the separation device. The yield for different cell cycle phases was obtained from signals of the DNA content and the EGFP signal, which marked the diESCs [Figs. 2(f) and 2(g)]. We estimated that a 7.7-fold (3.6 s.d.) enrichment for haESCs can be achieved by a single application of the separation procedure on average. The yield of the separation device decreased steadily from haploid G1 to diploid G2/M phase cells [Fig. 2(g)]. The observed yields were largely independent of different input ratios, indicating that there is no systematic effect of the haploid content of the input mixture on the separation performance [colored points, Fig. 2(g)]. Propidium iodide staining showed an average of 14.8% (14.0% s.d., n = 15) dead cells after passing the separation device [Fig. 2(h)]. Thus, cell viability is comparable or better than that of flow sorted cells, which showed an average of 21.9% (9.6% s.d., n = 9) of dead cells. The distribution of cell sizes is shifted towards smaller cells [Fig. 2(i)] with a size threshold, which likely corresponds to the size cutoff of the 8 *μ*m membrane for mouse ESCs and effectively excludes diploid ESCs.

## DISCUSSION

Our method for purifying and cultivating haESCs offers advantages over FACS based protocols. First, handling of potentially mutagenic nucleic acid stains can be avoided. Consequently, incubation time for stains and sorting time can be eliminated. It is foreseeable that this method can be parallelized to multiwell formats. This method avoids the use of chemical culture supplements, which could lead to unintended effects in some circumstances, and is compatible with a wide range of culture media. Cell viability measurements indicate that comparable or better viability is obtained compared to flow cytometric cell sorting. Taken together, these characteristics simplify the handling of haESC cultures and lead to considerable time savings, making haESCs accessible to many laboratories without a requirement for a cell sorter.

Notably, we find that minimizing force during passage through micrometer pores ensures high cell viability, facilitating future applications to human haESC cultures^7^ and to small cell numbers. It is conceivable to isolate haploid cells from early passage cultures, when establishing haESC lines, or isolated colonies after genetic modification. Practically, ESC colonies from haploid blastocysts or antibiotic selection often consist of a large fraction of already diploidized cells, and the small cell number often limits the possibility for flow cytometric cell sorting. The facility to separate haESCs before expansion can help to obtain haESC lines more efficiently. In addition to haESCs, enrichment of cells for specific cell cycle phases is also feasible by combining several membranes of different apertures. Calibration of aperture size and force-free separation makes our method applicable for cell purification in many areas that require high viability and rapid processing. It is enticing to speculate on integration of this method into single cell workflows in the future.

In summary, we report a new force-free size separation method for mammalian cells. The robustness of our method allows it to be incorporated into routine cell culture workflows and further does not require specialized instrumentation. We demonstrate the application for maintaining haploid cell cultures with a simpler workflow than flow cytometric or centrifugal elutriation techniques. In addition, the technique has potential for adaptation for cell cycle phase enrichment of mammalian cell cultures.

## SUPPLEMENTARY MATERIAL

See supplementary material for an instructional video showing the usage of the separation device and the flow cytometric gating strategy for separating cell cycle and ploidy fractions of the mixture experiment. The effect of sequential loading of media to the separation device on cell purity and yield is shown in Fig. 2 in the supplementary material.

## References

[1] L. H. Hartwell , R. K. Mortimer , J. Culotti , and M. Culotti , Genetics 74, 267–286 (1973). 17248617 10.1093/genetics/74.2.267 PMC1212945

[2] B. P. Brandhorst and G. E. Corley-Smith , Methods Mol. Biol. 254, 255–270 (2004). 15041767 10.1385/1-59259-741-6:255

[3] E. Wiellette , Y. Grinblat , M. Austen , E. Hirsinger , A. Amsterdam , C. Walker , M. Westerfield , and H. Sive , Genesis 40, 231–240 (2004). 10.1002/gene.20090 15593329

[4] M. Leeb and A. Wutz , Nature 479, 131–134 (2011). 10.1038/nature10448 21900896 PMC3209452

[5] M. Hirabayashi , H. Hara , T. Goto , A. Takizawa , M. R. Dwinell , T. Yamanaka , S. Hochi , and H. Nakauchi , J. Reprod. Dev. (published online). 10.1262/jrd.2017-074 PMC5735273 28824040

[6] H. Yang , Z. Liu , Y. Ma , C. Zhong , Q. Yin , C. Zhou , L. Shi , Y. Cai , H. Zhao , H. Wang , F. Tang , Y. Wang , C. Zhang , X. Y. Liu , D. Lai , Y. Jin , Q. Sun , and J. Li , Cell Res. 23(10), 1187–1200 (2013). 10.1038/cr.2013.93 23856644 PMC3790242

[7] I. Sagi , G. Chia , T. Golan-Lev , M. Peretz , U. Weissbein , L. Sui , M. V. Sauer , O. Yanuka , D. Egli , and N. Benvenisty , Nature 532, 107–111 (2016). 10.1038/nature17408 26982723

[8] A. Monfort , G. Di Minin , A. Postlmayr , R. Freimann , F. Arieti , S. Thore , and A. Wutz , Cell Rep. 12(4), 554–561 (2015). 10.1016/j.celrep.2015.06.067 26190100 PMC4530576

[9] J. V. Forment , M. Herzog , J. Coates , T. Konopka , B. V. Gapp , S. M. Nijman , D. J. Adams , T. M. Keane , and S. P. Jackson , Nat. Chem. Biol. 13(1), 12–14 (2017). 10.1038/nchembio.2226 27820796 PMC5164930

[10] V. A. Blomen , P. Májek , L. T. Jae , J. W. Bigenzahn , J. Nieuwenhuis , J. Staring , R. Sacco , F. R. van Diemen , N. Olk , A. Stukalov , C. Marceau , H. Janssen , J. E. Carette , K. L. Bennett , J. Colinge , G. Superti-Furga , and T. R. Brummelkamp , Science 350, 1092–1096 (2015). 10.1126/science.aac7557 26472760

[11] J. E. Carette , M. Raaben , A. C. Wong , A. S. Herbert , G. Obernosterer , N. Mulherkar , A. I. Kuehne , P. J. Kranzusch , A. M. Griffin , and G. Ruthel , Nature 477(7364), 340 (2011). 10.1038/nature10348 21866103 PMC3175325

[12] T. Wang , K. Birsoy , N. W. Hughes , K. M. Krupczak , Y. Post , J. J. Wei , E. S. Lander , and D. M. Sabatini , Science 350(6264), 1096–1101 (2015). 10.1126/science.aac7041 26472758 PMC4662922

[13] A. Y. Chen , C. Yu , B. Gatto , and L. F. Liu , Proc. Natl. Acad. Sci. U.S.A. 90(17), 8131–8135 (1993). 10.1073/pnas.90.17.8131 7690143 PMC47302

[14] K. Wojcik and J. W. Dobrucki , Cytometry, Part A 73A(6), 555–562 (2008). 10.1002/cyto.a.20573 18459157

[15] A. Guo , S. Huang , J. Yu , H. Wang , H. Li , G. Pei , and L. Shen , Stem Cell Rep. 8(5), 1124–1134 (2017). 10.1016/j.stemcr.2017.03.025 PMC5425685 28457886

[16] S. Takahashi , J. Lee , T. Kohda , A. Matsuzawa , M. Kawasumi , M. Kanai-Azuma , T. Kaneko-Ishino , and F. Ishino , Development 141, 3842–3847 (2014). 10.1242/dev.110726 25252944

[17] Z.-Q. He , B.-L. Xia , Y.-K. Wang , J. Li , G.-H. Feng , L.-L. Zhang , Y.-H. Li , H.-F. Wan , T.-D. Li , K. Xu , X.-W. Yuan , Y.-F. Li , X.-X. Zhang , Y. Zhang , L. Wang , W. Li , and Q. Zhou , Cell Rep. 20(9), 2227–2237 (2017). 10.1016/j.celrep.2017.07.081 28854370

[18] R. Freimann , S. Kramer , A. Böhmler , and A. Wutz , Biospektrum 20(4), 416–418 (2014). 10.1007/s12268-014-0458-6

[19] D. N. Simon and K. L. Wilson , Nat. Rev. Mol. Cell Biol. 12(11), 695–708 (2011). 10.1038/nrm3207 21971041

[20] W. Zhang , K. Kai , D. S. Choi , T. Iwamoto , Y. H. Nguyen , H. Wong , M. D. Landis , N. T. Ueno , J. Chang , and L. Qin , Proc. Natl. Acad. Sci. U.S.A. 109(46), 18707–18712 (2012). 10.1073/pnas.1209893109 23112172 PMC3503214

[21] Q.-L. Ying , J. Wray , J. Nichols , L. Batlle-Morera , B. Doble , J. Woodgett , P. Cohen , and A. Smith , Nature 453(7194), 519–523 (2008). 10.1038/nature06968 18497825 PMC5328678

[22] M. Leeb , R. Walker , B. Mansfield , J. Nichols , A. Smith , and A. Wutz , Development 139(18), 3301–3305 (2012). 10.1242/dev.083675 22912412 PMC3424041

